# Oridonin Alters Hepatic Urea Cycle via Gut Microbiota and Protects against Acetaminophen-Induced Liver Injury

**DOI:** 10.1155/2021/3259238

**Published:** 2021-10-21

**Authors:** Mu-keng Hong, Hai-hua Liu, Gui-hong Chen, Jun-qing Zhu, Song-yuan Zheng, Di Zhao, Jianxing Diao, Hui Jia, Ding-ding Zhang, Shi-xian Chen, Lei Gao, Juan Li

**Affiliations:** ^1^Department of Rheumatic & TCM Medical Center, Nanfang Hospital, Southern Medical University, Guangzhou, China; ^2^Department of Traditional Chinese Internal Medicine, School of Traditional Chinese Medical, Southern Medical University, Guangzhou, China; ^3^Department of Obstetrics and Gynecology, Nanfang Hospital, Southern Medical University, Guangzhou, China

## Abstract

Acetaminophen (APAP) hepatotoxicity is the leading cause of acute liver failure in the western world. Oridonin (OD), which is the major active ingredient of the traditional Chinese medicine *Rabdosia rubescens*, reportedly exerts anti-inflammatory and antioxidative effects. Here, we first find that OD protects against APAP-induced hepatotoxicity. The results of hepatic tissue-associated RNA-seq and metabolomics showed that the protective effects of OD were dependent upon urea cycle regulation. And such regulation of OD is gut microbiota partly dependent, as demonstrated by fecal microbiota transplantation (FMT). Furthermore, using 16S rRNA sequencing, we determined that OD significantly enriched intestinal *Bacteroides vulgatus*, which activated the nuclear factor erythroid 2-related factor 2 (Nrf2) pathway to regulate redox homeostasis against APAP by urea cycle. In conclusion, our study suggests that the *Bacteroides vulgatus*-urea cycle-Nrf2 axis may be a potential target for reducing APAP-induced liver injury, which is altered by OD.

## 1. Introduction

Acetaminophen (APAP) is a common analgesic and antipyretic drug. However, an APAP overdose can lead to severe hepatotoxicity, acute liver failure (ALF), and death [1]. Under normal conditions, APAP is predominantly metabolized via sulfation and glucuronidation in the liver. A small amount of the drug is metabolized by cytochrome P450 enzymes into N-acetyl-p-benzoquinoneimine (NAPQI), which is mainly detoxified by glutathione (GSH) [2, 3]. An overdose of APAP results in excess NAPQI, which exhausts hepatic GSH, leading to mitochondrial dysfunction and damage, and thereby hepatocyte necrosis and ALF [4, 5].

The urea cycle, which is exclusively located in the liver, the central organ for metabolism and detoxification, is a vital process involving 5 enzymes (Cps1, Otc, Ass1, Asl, and Arg1) that convert the highly toxic metabolic byproduct, ammonia, into urea, following amino acid metabolism [6, 7]. Urea cycle dysregulation has been reported in some liver diseases and causes high mortality, such as in hepatitis B virus-related liver failure and nonalcoholic fatty liver disease [8, 9]. Moreover, urea cycle-related amino acids arginine-ornithine homeostasis plays a crucial role for liver injury by adaptive immune response regulation [9]. Therefore, it was necessary to assess the influence of the urea cycle on APAP-induced liver injury.

Increasing studies suggest that the gut microbiota might play a crucial role in hepatotoxicity induced by an overdose of APAP [10]. Antibiotic-treated mice or germ-free mice can attenuate APAP hepatotoxicity [11]. Gut-resident lactobacilli protect against liver injury and reduce the effects of APAP overdoses by activating hepatic Nrf2 [12]. Furthermore, recent studies suggest that the gut microbiota mediates the hepatic urea cycle, amino acid metabolism, and bile acid biosynthesis reprogramming against hepatotoxicity [13]. Thus, the gut microbiota is essential for hepatic detoxification and modulating the host metabolism pathway through the gut microbiota might be an effective therapeutic strategy against APAP hepatotoxicity.

Oridonin (OD), the main component of *Rabdosia rubescens*, has been described as a hepatoprotective agent in many studies, most of which have focused on liver injury, liver cancer, and hepatic fibrosis involving antiapoptosis and antioxidative effects [14–17]. However, whether OD can ameliorate APAP-induced liver injury and its underlying mechanisms remains unclear. Herein, we report that OD alleviates APAP-induced liver injury. By integration of transcriptome analysis and hepatic metabolomics, we revealed that such protective effects were dependent upon urea cycle regulation via the activation of the Nrf2 pathway. Furthermore, we determined that OD modulated the gut microbiota against APAP hepatotoxicity and significantly enriched intestinal *B. vulgatus*, which alleviates APAP-induced liver injury through the urea cycle. Collectively, our study indicated that OD alleviated APAP-induced injury by altering the gut microbiota.

## 2. Materials and Methods

### 2.1. Animals and Drug Administration

Eight-week-old male C57BL/6 mice were obtained from the SPF Biotechnology Company (Beijing, China). *Nrf2^−/−^* mice were purchased from the Jackson Laboratory (USA). Mice were gavaged with 300 mg/kg APAP dissolved in phosphate-buffered saline (PBS). OD was purchased from Shanghai Yuanye Bio-Technology (Shanghai, China) and was dissolved in distilled water. For OD treatment, mice were injected intraperitoneally with 20 mg/kg OD 1 h after APAP administration. All mice were sacrificed after 24 h for the following experiments. All animal experimental procedures were approved by the local Animal Care and Use Committee of the Southern Medical University.

### 2.2. RNA Sequencing

RNA from liver tissues was isolated and checked for integrity and quantity using an RNA Nano 6000 Assay Kit (Agilent Technologies, Santa Clara, CA, USA, production number: 5067-1511) with the Bioanalyzer 2100 system (Agilent Technologies, Santa Clara, CA, USA). Following mRNA purification and cDNA synthesis, RNA sequencing libraries were generated using a NEBNext Ultra™ RNA Library Prep Kit from Illumina (New England Biolabs, Ipswich, MA, USA) following the manufacturer's protocol. After estimating the quality of the libraries, the samples were sequenced on an Illumina NovaSeq platform (San Diego, CA, USA), and 150 bp paired-end reads were generated.

### 2.3. Fecal DNA Isolation and 16S rRNA Amplicon Sequencing

Briefly, the cecal content DNA was extracted using the MinkaGene Tissue DNA kit (Magigene, Guangdong, China). The V3-V4 region of bacterial 16S rRNA was amplified using specific barcoded primers, and all PCRs were conducted using Phusion High-Fidelity PCR Master Mix (New England Biolabs). The PCR products were purified using a Qiagen Gel Extraction Kit (Qiagen, Hilden, Germany). Sequencing libraries were generated using a TruSeq DNA PCR-Free Sample Preparation Kit (Illumina, USA) according to the manufacturer's instructions. The quality of DNA libraries was assessed using a Qubit@ 2.0 Fluorometer (Thermo Fisher Scientific, Waltham, MA, USA) and an Agilent Bioanalyzer 2100 system. Finally, the samples were sequenced on an Illumina NovaSeq platform.

### 2.4. Fecal Microbiota Transplantation (FMT)

FMT was performed according to an established protocol [18]. In brief, recipient mice were orally gavaged with nonabsorbable antibiotics, including vancomycin (100 mg/kg), neomycin sulfate (200 mg/kg), metronidazole (200 mg/kg), and ampicillin (200 mg/kg), once daily for 5 d. Fresh cecal content collected from donor mice (APAP group, APAP+OD group) was diluted in PBS at 0.125 g/mL. Recipient mice were fed transplant material for 3 d, following which mice were gavaged with 300 mg/kg APAP and sacrificed 24 h after APAP was administered.

### 2.5. Targeted Metabolomics for Amino Acids

The samples (100 mg) were resuspended in liquid nitrogen and diluted with water. Then, 50 *μ*L of the 100-fold diluted sample was taken and homogenized with 200 *μ*L of acetonitrile/methanol (1 : 1), which contained mixed internal standards. The sample was left on ice for 30 min and centrifuged at 12000 rpm for 10 min at 4°C to remove the protein. Finally, the supernatant was injected into the LC-MS/MS system for analysis. An ultrahigh-performance liquid chromatography coupled with a tandem mass spectrometry (UHPLC-MS/MS) system (ExionLC™ AD UHPLC-QTRAP 6500+; AB SCIEX Corp., Boston, MA, USA) was used to quantify amino acids by Novogene Co., Ltd. (Beijing, China). The separation was performed on an ACQUITY UPLC BEH Amide column (2.1 × 100 mm, 1.7 *μ*m), which was maintained at 50°C. The mobile phase, consisting of 0.1% formic acid in water with 5 mM ammonium acetate (solvent A) and 0.1% formic acid in acetonitrile (solvent B), was delivered at a flow rate of 0.30 mL/min. The solvent gradient was set as follows: initial, 80% B, 0.5 min; 80–70% B, 2 min; 70–45% B, 4 min; 45–80% B, 6.01 min; and 80% B, 9 min. The mass spectrometer was operated in positive multiple reaction mode. Parameters were as follows: ion spray voltage (5 500 V), curtain gas (35 psi), ion source temperature (550°C), and ion source gas of 1 (50 psi) and 2 (65 psi). To detect dysregulated amino acids, one-way analysis of variance (ANOVA) was first used to detect the differences among groups, and a post hoc test (Tukey's honestly significant difference; HSD) was used to perform multiple comparisons. An unpaired *t*-test was used to detect dysregulated metabolites between the two groups.

### 2.6. Quantification and Statistical Analysis

All continuous variables were compared between groups using Student's *t*-test. If there were more than two groups, one-way ANOVA was first used, and then Tukey's HSD method was used. All statistical analyses were performed using GraphPad software. The data are presented as the mean ± standard error of mean (SEM). Statistical significance was described as ^∗^*p* < 0.05, ^∗∗^*p* < 0.01, or ^∗∗∗^*p* < 0.001.

For further details regarding the materials used, please refer to the supplementary information (available here).

## 3. Results

### 3.1. OD Mitigates APAP-Induced Liver Injury

**Figure 1:**
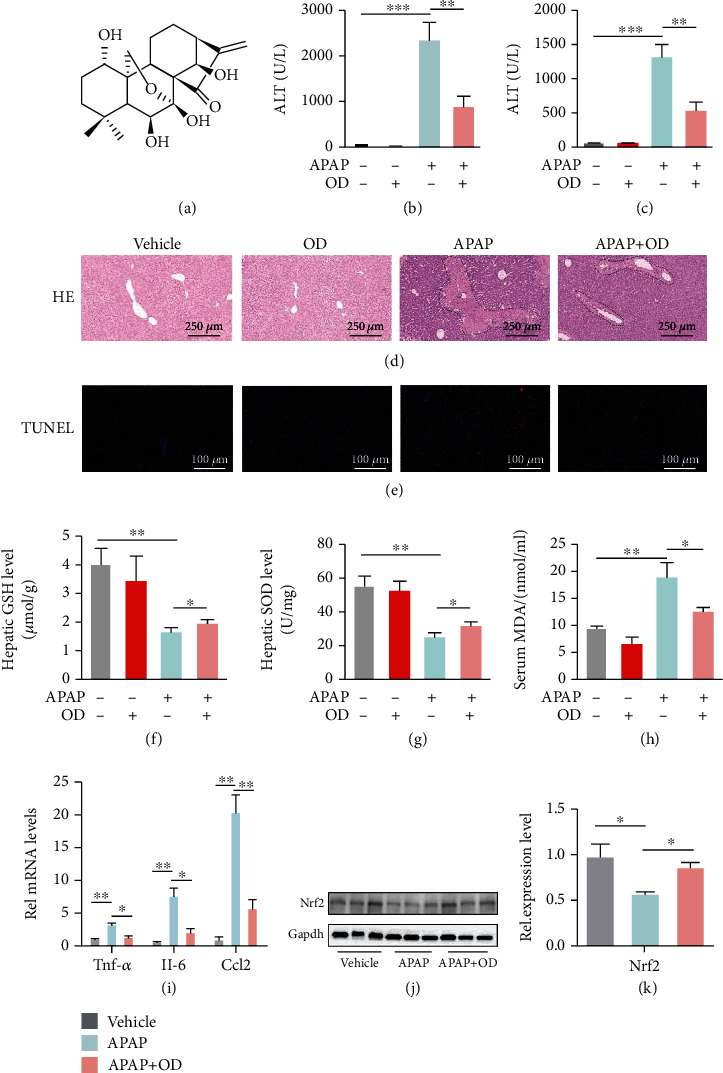
Oridonin (OD) alleviates APAP-induced liver injury. (a) OD structure. (b, c) Serum ALT and AST levels (*n* = 10). (d, e) Representative H&E and TUNEL staining images of liver tissue in each group. (f, g) Hepatic GSH and SOD in each group (*n* = 6–8). (h) Levels of serum MDA (*n* = 6–9). (i) mRNA levels of cytokines and chemokines in the liver (*n* = 6). (j, k) Western blot for Nrf2. The data are presented as the mean ± standard error of mean (SEM). ^∗^*p* < 0.05, ^∗∗^*p* < 0.01, and ^∗∗∗^*p* < 0.001.

We evaluated whether OD could protect the liver from APAP-induced injury (Figure 1(a)). Mice were treated with 20 mg/kg OD 1 h after administering APAP. OD attenuated APAP hepatotoxicity in a dose-dependent manner, and 20 mg/kg could attenuate the syndrome induced by APAP according to ALT and AST levels (Figure S1A-B). At 24 h after administering APAP, serum ALT and AST levels increased significantly, leading to centrilobular necrosis (Figures 1(b)–1(d)). Compared to that in the APAP-treated group, liver injury was remarkably attenuated in the OD-treated group (Figures 1(b)–1(d)). TUNEL staining confirmed these results (Figure 1(e)).

Increasing studies demonstrated that CYP2E1 converted APAP to NAPQI and caused oxidative stress [19]. To investigate the potential for OD to affect drug metabolism, we detected hepatic CYP2E1 expression. APAP administration upregulated CYP2E1 expression, while OD administration could not significantly affect CYP2E1 protein expression (Figure S2A-B). To assess hepatic oxidative stress, we determined hepatic GSH and SOD. APAP treatment significantly decreased liver GSH and SOD, whereas GSH and SOD in the OD-treated group were obviously improved (Figures 1(f) and 1(g)). Furthermore, the level of serum malondialdehyde, a product of oxidative damage, indicated that OD diminished oxidative stress caused by APAP overdose (Figure 1(h)). Additionally, OD also reduced the hepatic expression of inflammatory factors (Figure 1(i)). Nrf2 plays a pivotal role in redox homeostasis and APAP-induced hepatotoxicity, and Nrf2-mediated gene regulation is necessary for hepatic GSH synthesis, detoxification, and transport [20]. Nrf2 in the APAP group was significantly decreased compared to those in the vehicle group. The expression levels of all these proteins were effectively increased by OD (Figures 1(j) and 1(k)). These data demonstrated that OD exhibits robust efficacy and antioxidative effect against APAP hepatotoxicity.

### 3.2. OD Attenuates Urea Cycle Dysregulation against APAP Hepatotoxicity

**Figure 2:**
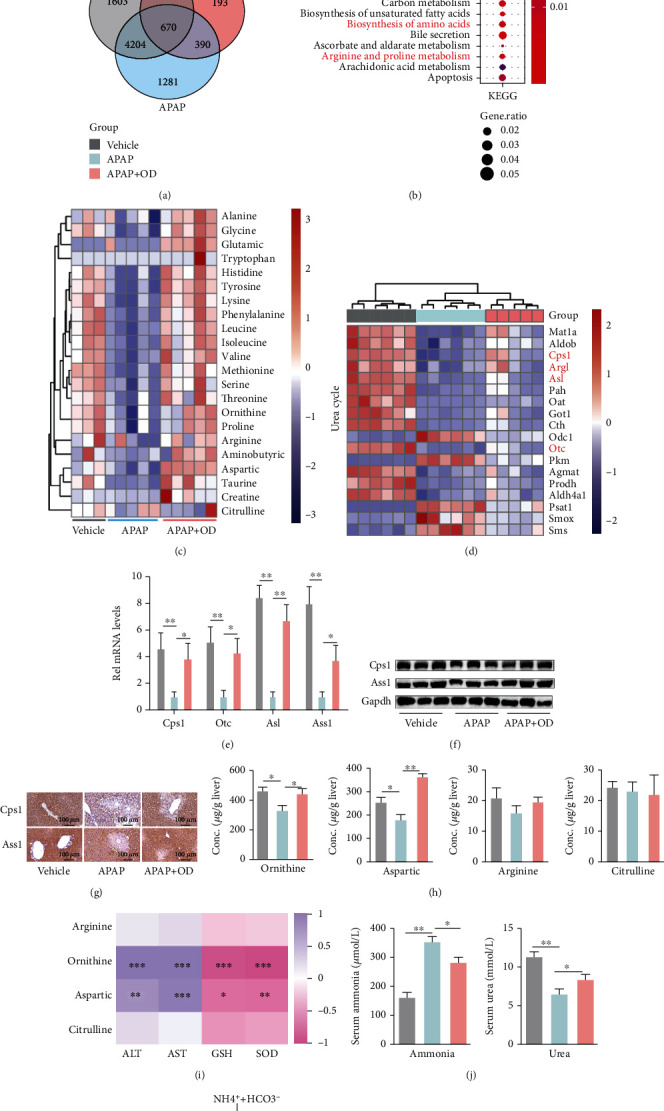
Oridonin (OD) attenuates urea cycle dysregulation against APAP hepatotoxicity. (a) Venn diagram of the unique and commonly changed genes in each group (*n* = 5–6). (b) KEGG pathway analysis of rescued genes (*n* = 5–6). Red boxes highlight pathways involved in amino acid metabolism. (c) The expression level of different amino acids in each group (*n* = 3–5). (d) Heatmap of expression of genes involved in urea cycle pathways in RNA-seq analysis. (e) qPCR analysis of urea cycle-related genes (*n* = 6). (f) Western blot analysis of Cps1 and Ass1 (*n* = 3). (g) Representative images of Cps1 and Ass1 immunohistochemical staining of liver tissues. (h) Urea cycle-associated metabolites by LC-MS (*n* = 3-5). (i) Correlations between hepatic amino acids and liver injury parameters. (j) Serum ammonia and urea levels (*n* = 6-8). (k) Schematic diagram showing the urea cycle. The data are presented as the mean ± standard error of mean (SEM). ^∗^*p* < 0.05, ^∗∗^*p* < 0.01.

To further explore the mechanism underlying the protective effects of OD, we performed transcriptome analysis via RNA sequencing among the three groups (vehicle, APAP, and APAP+OD). After treatment with OD, the expression levels of 1327 of 1451 genes were rescued (775 upregulated genes in APAP-treated mice were downregulated by OD treatment, and 572 downregulated genes in APAP-treated mice were upregulated by OD treatment) (Figure 2(a) and Figure S3A). Then, we performed pathway enrichment analysis of these rescued genes using clusterProfiler [21]. Most of the significant pathways were related to amino acid metabolism and oxidative stress (Figure 2(b) and Figure S3B). Meanwhile, by performing amino acid metabolomics, APAP cause hepatic amino acid metabolism dysregulation and OD relieved this dysfunction (Figure 2(c) and Figure S3C).

The urea cycle pathway is associated with amino acid and nucleotide metabolic pathways and represents a network of systemic nitrogen metabolism [9]. According to the transcriptome results, the urea cycle was sharply suppressed by APAP but was recovered by OD (Figure 2(d) and Figure S3D). Consistent with the transcriptome results, the mRNA levels of urea cycle-related gene and protein levels were suppressed by APAP and were improved by OD administration (Figures 2(e)–2(g)). The urea cycle-related amino acids especially ornithine were reduced in the APAP group compared with the control and OD groups (Figure 2(h)). Pearson's correlation analysis showed significant correlations between these amino acids and impaired hepatic functions and oxidative stress and particularly that of ornithine (Figure 2(i)). Moreover, accompanied by urea cycle disruption, APAP suppressed serum urea production and increased serum ammonia, whereas OD promoted urea production and decreased serum ammonia (Figure 2(j)). Taken together, APAP appears to disrupt the hepatic urea cycle, whereas OD appears to attenuate the urea cycle dysfunction caused by APAP (Figure 2(k)).

### 3.3. OD Attenuates APAP-Induced Liver Injury in a Urea Cycle-Associated Manner

**Figure 3:**
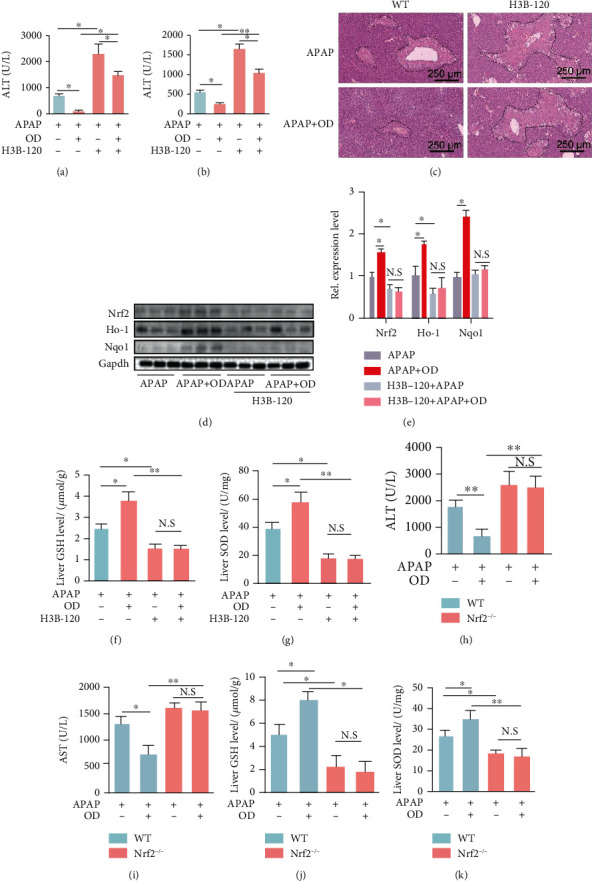
Oridonin (OD) attenuates APAP-induced liver injury in a urea cycle-associated manner. (a, b) ALT and AST in different groups. (c) Representative H&E images. (d, e) Western blot analysis of Nrf2, HO-1, and Nqo1 (*n* = 3). Hepatic GSH (f) and SOD (g) in different groups (*n* = 5-6). (h, i) Serum ALT and AST levels in wild-type (WT) and Nrf2^−/−^ mice after APAP administration of vehicle or OD treatment (*n* = 4–7). Hepatic GSH (j) and SOD (k) in different groups. The data are presented as the mean ± standard error of mean (SEM). ^∗^*p* < 0.05; ^∗∗^*p* < 0.01; N.S: not statistically significant.

To further examine whether OD alleviated APAP hepatotoxicity in a urea cycle-dependent manner, we used H3B-120 (a potent Cps1 inhibitor) to inhibit the urea cycle [22]. Mice were pretreated with H3B-120 (20 mg/kg) 12 h before APAP administration. H3B-120 administration significantly increased serum ALT and AST levels and diminished the protective effects of OD, which indicated that OD attenuated APAP-induced liver injury in a urea cycle-associated manner (Figures 3(a) and 3(b)). Liver histology also confirmed these results (Figure 3(c)). Meanwhile, the APAP+H3B-120 group showed significantly higher serum ALT and AST levels compared with the APAP+OD+H3B-120 group (Figures 3(a) and 3(b)). These data suggested that the protective effect of OD was partly dependent on urea cycle, and other mechanisms need further investigation.

Above data suggested that OD exhibits an antioxidative effect against APAP hepatotoxicity through Nrf2 pathway activation (Figures 1(j) and 1(k)). Additionally, increasing studies suggested that urea cycle is an important regulator of hepatic redox homeostasis. We further explore the relationship between the urea cycle and Nrf2 pathway. Consistent with impaired hepatic functions, H3B-120 administration also significantly inhibited expression of Nrf2 and relative antioxidative genes (Ho-1, Nqo1) (Figures 3(d) and 3(e)). Meanwhile, Nrf2 pathway activation by OD was diminished after urea cycle inhibition (Figures 3(d) and 3(e)). Moreover, the increases of hepatic GSH and SOD by OD administration were markedly exhausted by H3B-120 (Figures 3(f) and 3(g)). Thus, we predicated that OD modulated the urea cycle to relieve oxidative stress caused by APAP in an Nrf2-dependent manner. In contrast to WT mice, serum ALT and AST levels were increased and hepatic GSH and SOD levels were decreased in *Nrf2^−/−^* mice (Figures 3(h)–3(k)). Monitoring of serum ALT and AST levels and hepatic GSH and SOD levels indicated no difference in liver injury and oxidative stress between the control and OD groups upon deletion of *Nrf2* in mice (Figures 3(h)–3(k)). Taken together, these results suggested that OD altered the urea cycle to alleviate APAP hepatotoxicity in an Nrf2-dependent manner.

### 3.4. OD Alters Urea Cycle and Attenuates APAP-Induced Liver Injury in a Gut Microbiota-Associated Manner

**Figure 4:**
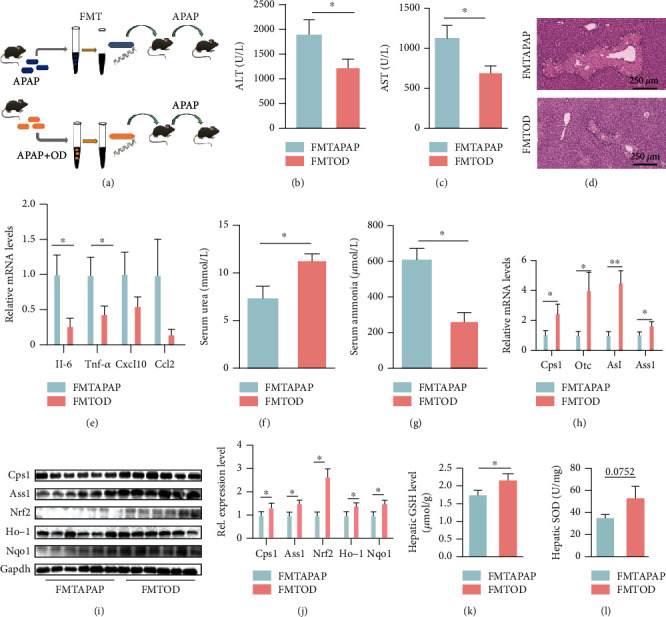
Oridonin (OD) alters urea cycle and attenuates APAP-induced liver injury in a gut microbiota-associated manner. Mice were pretreated with antibiotics for one week and received the cecal contents from OD-treated mice or APAP-treated mice before APAP administration. (a) FMT experimental design. (b, c) Serum ALT and AST levels (*n* = 8). (d) Representative H&E images. (e) mRNA levels of cytokines and chemokines in the liver (*n* = 8). (f, g) Serum ammonia and urea levels (*n* = 6). (h) qPCR analysis of urea cycle-related genes (*n* = 6). (i, j) Western blot analysis of the urea cycle and Nrf2 pathway. (k, l) Hepatic GSH and SOD in both groups (*n* = 8). The data are presented as the mean ± standard error of mean (SEM). ^∗^*p* < 0.05; ^∗∗^*p* < 0.01.

Recent studies have demonstrated that the gut microbiome is crucial for redox homeostasis and APAP hepatotoxicity. Considering that the low bioavailability of OD suggests that it may act as a potential substrate for gut microbiota and OD could not directly inhibit hepatocyte apoptosis in vitro (Figure S4A-B) [23], we hypothesized that the protective effect of OD might be dependent on the gut microbiome. To further investigate the contribution of the gut microbiome to the protective effect of OD, we performed an FMT experiment. Following 1 week of antibiotic treatment, recipient mice were randomly divided into 2 groups, which were subjected to microbiota transfer from either APAP-treated mice or OD-posttreated (APAP and APAP+OD) mice (Figure 4(a)). The levels of serum ALT and AST were notably decreased in the group that received the cecal contents from OD-treated mice (FMTOD) compared to those in the group that received the microbiota from APAP-treated mice (FMTAPAP) (Figures 4(b) and 4(c)). Liver histology indicated that FMTOD mice consistently exhibited smaller hepatic necrosis areas compared with those in FMTAPAP mice (Figure 4(d)). Additionally, the hepatic expression of inflammatory factors was decreased in FMTOD mice (Figure 4(e)).

Furthermore, we test whether the gut microbiota mediated OD-induced urea cycle-Nrf2 pathway activation. FMTAPAP mice showed serum ammonia accumulation and urea production disruption compared to those in FMTOD mice (Figures 4(f) and 4(g)). Moreover, the FMTOD group exhibited significantly higher gene expression in relation to the urea cycle, compared to that in the FMTAPAP group (Figure 4(h)). The mRNA results were confirmed by the protein levels of Cps1 and Ass1 (Figures 4(i) and 4(j)). Meanwhile, with urea cycle upregulation, the Nrf2 pathway was significantly activated in the FMTOD group (Figures 4(i) and 4(j)). In line with the expression changes, the levels of GSH and SOD in the FMTOD group were higher than those in the FMTAPAP group (Figures 4(k) and 4(l)).

### 3.5. OD Alters APAP-Induced Gut Dysbiosis and Enriches *B. vulgatus*

**Figure 5:**
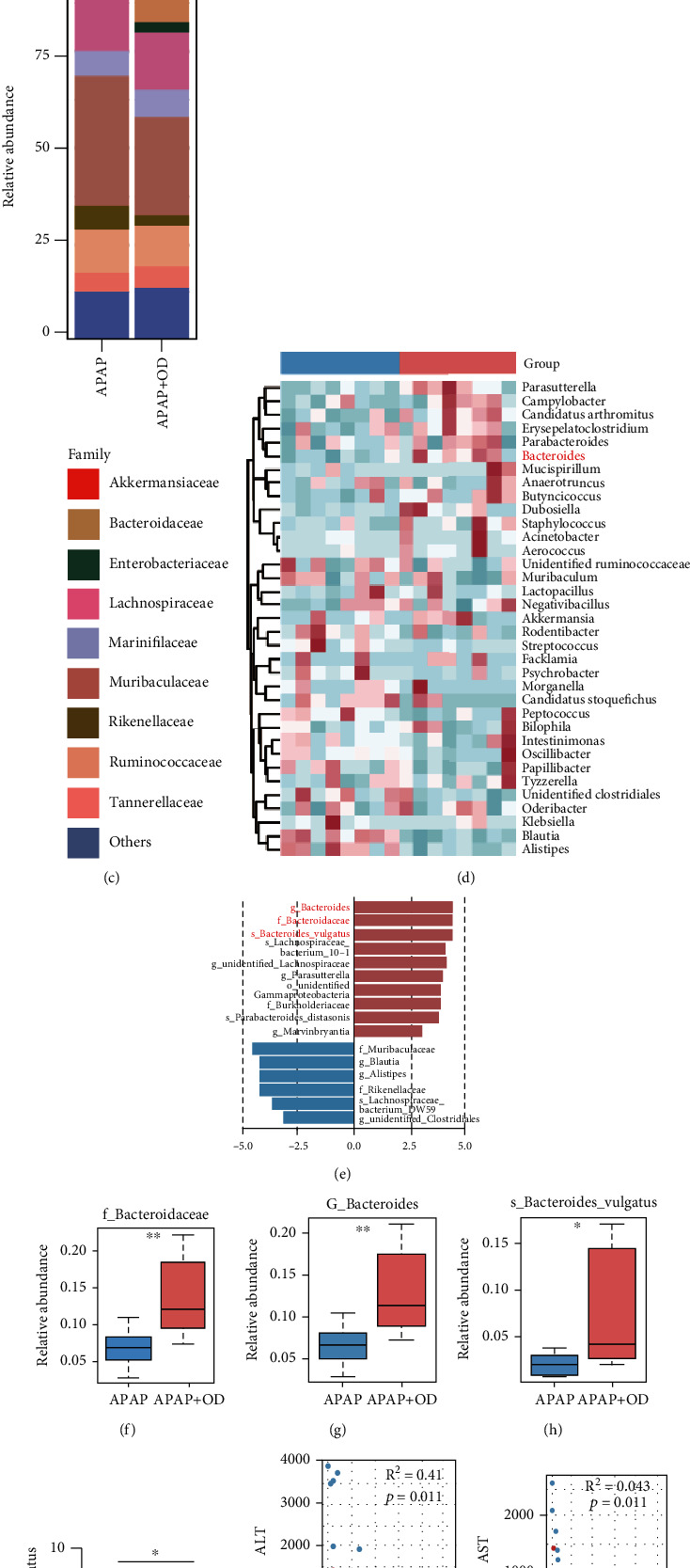
Oridonin (OD) alters APAP-induced gut dysbiosis and enriches *B. vulgatus*. Gut microbiota compositions of the APAP and APAP+OD groups were analyzed by 16S rRNA sequencing. (a) PCoA analysis of the gut microbiota based on the OTU data of the APAP and APAP+OD groups (*n* = 8). (b) The *beta*-diversity of each group (*n* = 8). (c) The relative abundance of bacteria at the family level (*n* = 8). (d) Heatmap of OTU analysis of each group (*n* = 8). (e) Discriminative taxa determined by Lefse between the APAP and APAP+OD groups (log_10_ LDA > 3.0) (*n* = 8). (f–h) Relative abundance of family (f), genus (g), and species (h) of *B. vulgatus* (*n* = 8). (i) qPCR analysis of *B. vulgatus* abundance. (j, k) Scatterplots show correlations between *B. vulgatus* abundance and ALT (j) and AST (k) levels. The data are presented as the mean ± standard error of mean (SEM). ^∗^*p* < 0.05; ^∗∗^*p* < 0.01.

Results in the current study showed that the protective effect of OD was strongly associated with the gut microbiota. Therefore, we performed 16S rRNA sequencing to examine the diversity of the intestinal microbiota community. Principal coordinate analysis (PCoA) and the beta-diversity revealed distinct microbiota composition clustering for the APAP and OD treatment groups (Figures 5(a) and 5(b)). The alpha-diversity values showed no significant difference between the groups, including Chao1 and observed operational taxonomic units (Figure S5A, B). Next, we observed that OD changed the gut microbiota composition at different taxonomic levels. Specially, at the family level, OD increased the abundance of *Akkermansiaceae* and *Bacteroidaceae* (Figure 5(c)). Moreover, at the genus level, the abundances of *Akkermansia* and *Bacteroides* were enriched in the OD groups, whereas *Alistipes* and *Blautia* were enriched in the APAP groups (Figure 5(d) and Figure S5C).

To further identify the specific bacterial phylotypes between the APAP and OD groups, we computed the linear discriminant analysis effect size and selected species based on a linear discriminant analysis score > 3 (Figure 5(e)). Gut microbiota changed substantially, and *B. vulgatus* was significantly enriched in response to the OD treatment (Figures 5(f)–5(h)), which the qPCR analysis confirmed (Figure 5(i)). The abundance of *B. vulgatus* was correlated with ALT and AST (Figures 5(j) and 5(k)). The OD treatment alleviated the reduction of the tight junction markers ZO-1 and occludin induced by APAP (Figure S5D-E). These results indicate that disruption of the gut barrier function and dysbiosis of the gut microbiota, induced by APAP, were dramatically ameliorated by OD treatment. The results suggest that OD can alter gut microbiota composition and substantially enrich *B. vulgatus*.

### 3.6. *B. vulgatus* Enrichment by OD Alters Urea Cycle Dysregulation against APAP Hepatotoxicity

**Figure 6:**
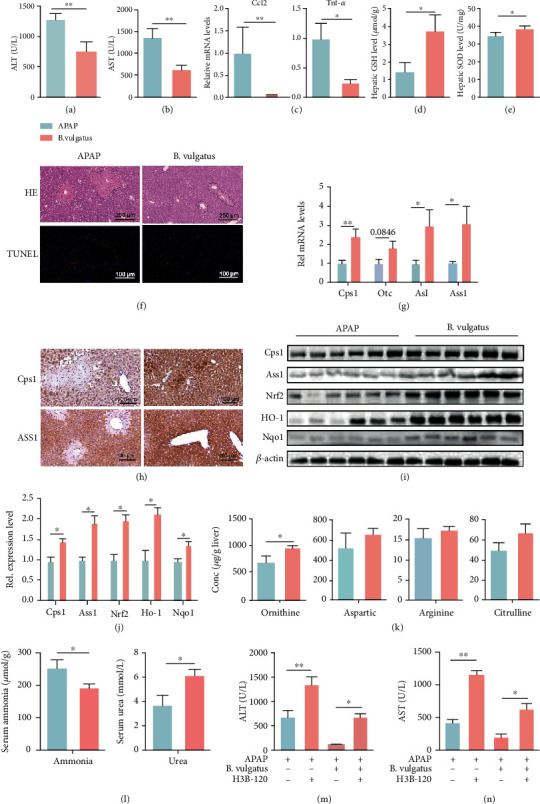
*B. vulgatus* enrichment by OD alters urea cycle dysregulation against APAP hepatotoxicity. (a, b) Serum ALT and AST levels in mice treated with vehicle or *B. vulgatus* prior to APAP treatment (*n* = 8). (c) mRNA levels of cytokines and chemokines in the liver (*n* = 6). (d, e) Hepatic GSH and SOD in both groups (*n* = 7). (f) Representative H&E and TUNEL staining images. (g) qPCR analysis of urea cycle-related genes (*n* = 6). (h) Representative images of Cps1 and Ass1 immunohistochemical staining of liver tissues. (i, j) Western blot analysis of urea cycle and Nrf2 pathway (*n* = 6). (k) Urea cycle-associated metabolites by LC-MS (*n* = 5-6). (l) Serum ammonia and urea levels (*n* = 6-8). (m, n) Serum ALT and AST levels in each group (*n* = 6). The data are presented as the mean ± standard error of mean (SEM). ^∗^*p* < 0.05, ^∗∗^*p* < 0.01.

*B. vulgatus* was substantially enriched by OD treatment. Next, we explored the potential association between *B. vulgatus* and APAP-induced liver injury. Mice were orally administered with either the vehicle (PBS) or *B. vulgatus* for 2 weeks prior to APAP treatment. Administering *B. vulgatus* did not cause a significant change in food intake and weight compared to the vehicle group (Figure S6A-B). Interestingly, gavage with *B. vulgatus* lowered serum ALT and AST levels, compared to those in the PBS-treated group (Figures 6(a) and 6(b)). Additionally, *B. vulgatus* also reduced hepatic expression of inflammatory factors (Figure 6(c)). Similarly, administering *B. vulgatus* remarkably improved hepatic GSH and SOD levels following APAP overdose (Figures 6(d) and 6(e)). These results were substantiated by H&E and TUNEL staining, which indicated that administering *B. vulgatus* greatly attenuated hepatic necrosis (Figure 6(f)). These results indicated that it was *B. vulgatus* that showed potential for protecting the liver from injury induced by APAP.

Notably, administering *B. vulgatus* significantly increased the mRNA expression of Cps1, Otc, Ass1, and Asl in the liver (Figure 6(g)). This change in expression levels was substantiated by protein levels (Figures 6(h)–6(j)). In line with results of OD administration, *B. vulgatus* increases the abundance of urea cycle-related amino acids especially ornithine (Figure 6(k) and Figure S4). Accompanied by urea cycle disruption, APAP suppressed serum urea production and increased serum ammonia, whereas *B. vulgatus* promoted urea production and decreased serum ammonia (Figure 6(l)). Moreover, urea cycle inhibition with H3B-120 increased ALT and AST levels and eliminated the protective effects of *B. vulgatus* (Figures 6(m) and 6(n)). These results indicated that *B. vulgatus* enrichment by OD attenuated APAP hepatotoxicity through the urea cycle-Nrf2 pathway.

## 4. Discussion

APAP hepatotoxicity is the most common cause of ALF in the western world [24]. Hence, a comprehensive understanding of the underlying mechanisms is urgently needed to develop more effective therapeutic approaches. Although OD evidently exerts anti-inflammatory and antiapoptotic effects on liver injury, there is no direct evidence supporting the notion that OD protects against APAP hepatotoxicity, and any underlying mechanism that might enable such protective activity has yet to be proposed, all of which limits its clinical application [15]. In the present study, we firstly demonstrated that OD could attenuate oxidative stress and liver injury against APAP hepatotoxicity. Moreover, by integrating the data pertaining to the gut microbiota, hepatic transcriptome, and metabolomics, we demonstrated that the protective effects of OD are partly dependent on the *B. vulgatus*-urea cycle-Nrf2 axis, which alleviated APAP-induced liver injury by reducing oxidative stress.

Urea cycle dysregulation has been reported in some liver diseases and causes high mortality [8, 9, 25]. Accumulating studies suggested that urea cycle was crucial for liver injury through adaptive immune response regulation and highly toxic metabolic byproduct elimination [26]. Presently, by performing transcriptome and metabolomics analysis, we proved that APAP sharply disrupted the hepatic amino acid metabolism pathway, especially the urea cycle, the crucial component of amino acid metabolism. More importantly, urea cycle inhibitor H3B-120 acutely exacerbated APAP hepatotoxicity and partly diminished the protective effect of OD. Therefore, urea cycle dysregulation could aggravate APAP-induced liver injury and OD attenuated APAP hepatotoxicity in a urea cycle-associated manner. Increasing studies suggested that the urea cycle is an important regulator of hepatic redox homeostasis [12]. Nrf2 is the key regulator for hepatic GSH, SOD, and other antioxidative element syntheses [19, 20]. We further explore the relationship between the urea cycle and Nrf2 pathway. Furthermore, urea cycle ablation inhibited the Nrf2 pathway, while OD activated the urea cycle and Nrf2 pathway. These findings suggested that urea cycle altered hepatic redox homeostasis through Nrf2 activation. Regulation on urea cycle might be a promising strategy for APAP hepatotoxicity therapy.

Accumulating studies have indicated a relationship between the gut microbiota and APAP-induced-liver injury [27, 28]. Additionally, recent studies have suggested that traditional Chinese medicine, as effective and potential prebiotics, could modulate the gut microbiome to prevent the development of various diseases [29]. Furthermore, it was previously demonstrated that OD could inhibit infection by *Salmonella pullorum* and protect intestinal health [30]. Thus, we speculated that the protective effect of OD might be associated with gut microbiota. According to FMT experiments, we found that OD alleviated APAP hepatotoxicity and regulated the urea cycle-Nrf2 pathway in a gut microbiota-associated manner. Of note, by performing 16S rRNA sequencing, we observed that treatment with OD led to the significant enrichment of *B. vulgatus* at the family-genus-species level.

Another finding of this study is that *B. vulgatus* could attenuate APAP-induced liver injury through the urea cycle-Nrf2 pathway. *B. vulgatus* is widely described as the main driver promoting the impact of branched-chain amino acids in host metabolism [31]. Branched-chain amino acid supplementation improves event-free survival in some advanced liver diseases [32, 33]. Additionally, recent studies showed that *B. vulgatus* suppresses proinflammatory immune responses by decreasing gut microbial lipopolysaccharide production [34]. *B. vulgatus*, a commensal bacterial species, reportedly inhibits colonization by pathogenic microbes [35]. These results indicate that *B. vulgatus* acts as a probiotic in the body. In this study, we further demonstrated that *B. vulgatus* could attenuate oxidative stress and liver injury caused by APAP. In line with results of OD administration, *B. vulgatus* could activate the urea cycle-Nrf2 pathway, while these effects were diminished by urea cycle inhibition. These results indicated that OD could attenuate APAP-induced liver injury partly in a gut microbiota-dependent manner, involving the enrichment of *B. vulgatus*.

In conclusion, the current results demonstrate that OD exerts a significant protective effect against APAP hepatotoxicity, which is partly dependent on urea cycle. Moreover, OD alters urea cycle against APAP hepatotoxicity through gut microbiota modulation involving the enrichment of *B. vulgatus.* Collectively, OD targets the *B. vulgatus*-urea cycle-Nrf2 axis and is a potential therapeutic agent for APAP-induced liver injury.

## Data Availability

The data that support the findings of this study are available from the corresponding authors upon reasonable request.

## Abbreviations

APAP: Acetaminophen
ALF: Acute liver failure
ABX: Antibiotic
FMT: Fecal microbiota transplantation
GSH: Glutathione
NAPQI: N-Acetyl-p-benzoquinoneimine
OD: Oridonin.

## Supplementary Materials

Supplementary Materials Table S1: primer sequences. Figure S1: OD attenuated APAP hepatotoxicity in a dose-dependent manner. Figure S2: OD does not significantly affect CYP2E1 protein expression. Figure S3: OD altered amino acid metabolism dysbiosis and urea cycle dysregulation against APAP hepatotoxicity. Figure S4: OD could not directly suppress hepatocyte apoptosis in vitro. Figure S5: OD alters gut dysbiosis against APAP hepatotoxicity. Figure S6: effect of *B. vulgatus* on body weight gain and food intake.
