# Supplementary material for: Oridonin Alters Hepatic Urea Cycle via Gut Microbiota and Protects against Acetaminophen-Induced Liver Injury
Source: Oxid Med Cell Longev. 2021 Oct 21;2021:3259238. doi: 10.1155/2021/3259238 (PMC8553473; doi:10.1155/2021/3259238)
Supplement: Supplementary Materials — Table S1: primer sequences. Figure S1: OD attenuated APAP hepatotoxicity in a dose-dependent manner. Figure S2: OD does not significantly affect CYP2E1 protein expression. Figure S3: OD altered amino acid metabolism dysbiosis and urea cycle dysregulation against APAP hepatotoxicity. Figure S4: OD could not directly suppress hepatocyte apoptosis in vitro. Figure S5: OD alters gut dysbiosis against APAP hepatotoxicity. Figure S6: effect of B. vulgatus on body weight gain and food intake. [file 3259238.f1.docx]

Supplementary Materials for

**Oridonin alters hepatic urea cycle via gut microbiota and protects against acetaminophen-induced liver injury**

Mu-keng Hong, Hai-hua Liu, Gui-hong Chen, Jun-qing Zhu, Song-yuan Zheng, Di Zhao, Jianxing Diao, Hui Jia, Ding-ding Zhang, Shi-xian Chen^*^, Lei Gao^*^, Juan Li^*^

*Corresponding author

**Materials and Methods**

**Measurement of ALT, AST, MDA, Ammonia, Urea, GSH and SOD contents**

The serum ALT, AST, MDA, Ammonia and Urea were detected by commercially available test kits (Nanjing Jiancheng Bioengineering Institute, Nanjing, China) according to the manufacturer’s instructions. GSH and SOD were measured from liver tissue using commercial kits (Nanjing Jiancheng Bioengineering Institute, Nanjing, China).

**Real time qPCR analysis.**

Total RNA were extracted from tissues using Trizol reagent (TransGen Biotech, Beijing, China) according to manufacturer’s protocol. We synthetized cDNA with RT Reagent kit (TransGen Biotech, Beijing, China) and performed quantitative real-time PCR on ABI PRISM 7500 real-time PCR system using SYBR Green mix (TransGen Biotech, Beijing, China). All primer sequences were shown in Table S1. 18s was used to normalize expression.

**Isolation of nuclear proteins and Western blotting**

We displayed nuclear proteins isolation by a Nuclear and Cytoplasmic Extraction Kit (Epizyme, Shanghai, China) following manufacturer’s protocol. For western blotting, liver tissues were lysed in RIPA buffer with protease and phosphatase inhibitors. And supernatants were collected by centrifugation at 12,000 rpm for 15 min at 4°C and BCA method was used to detected protein concentration. Equivalent proteins were separated by 10%-12.5% SDS-PAGE, transferred by PVDF membrane, and blotted with following antibodies: Caspase3, Caspase 8, Ripk1, Nrf2, Ho-1, iNOS, Cps1, Ass1, Gapdh (Proteinch, Chicago, USA).

**Histological analysis**

Livers and intestines tissues of mice were fixed in 10% formalin (Biosharp) and were embedded in paraffin wax. Then paraffin sections were stained with hematoxylin and eosin. For TUNEL staining experiment, we used commercial kit (KeyGene) according to the manufacturer’s protocol. For immunohistochemistry experiment, Zo-1, Occludin (Abcam, Cambridge, UK) and Cps1, Ass1 (Proteinch, Chicago, USA) were used according to the manufacturer’s recommendations.

**Bacterial cultures and preparation**

*B. vulgatus* (NO. 337471) was purchased from the BeNa Culture Collection (Beijing, China). All bacteria were stored in 20% glycerol at −80 °C. *B. vulgatus* was grown in LB broth. Prior to oral gavage, all live bacteria were collected, centrifuged at 3 000 × *g* for 5 min, and washed three times using PBS, following which, the supernatant was discarded. Live *B. vulgatus* bacteria were diluted in PBS to obtain a final concentration of 2×10^8^ CFU/mL for oral gavage. Mice were gavaged with live bacteria (0.2 mL) or PBS for 2 weeks.

**16S rRNA amplicon sequencing data analysis**

Paired-end reads was assigned to samples based on their unique barcode and truncated by cutting off the barcode and primer sequence and FLASH (V1.2.7) was used to merge reads. To obtain the high-quality clean tags, quality filtering on the raw tags were performed according to the quality control process of QIIME (V1.9.1). Chimera sequences were detected comparing with reference database (Silva database, <https://www.arb-silva.de/>) by the UCHIME algorithm, and then the chimera sequences were removed. Then the effective tags finally obtained. Sequencing analysis were performed by Uparse software. Sequences with more than 97% similarity were assigned to the same OTUs. Representative sequences were generated, singletons were removed, and a final OTU table was created. Representative sequences of OTUs were aligned using the Silva database for taxonomic classification. In order to study phylogenetic relationship of different OTUs, and the difference of the dominant species in different samples (groups), multiple sequence alignment were conducted using the MUSCLE software (Version 3.8.31, <http://www.drive5.com/muscle/>). OTUs abundance information were normalized using a standard of sequence number corresponding to the sample with the least sequences. Subsequent analysis of alpha diversity and beta diversity were all performed basing on this output normalized data.

Estimates of *alpha*-diversity were based on an evenly rarefied OTU abundance matrix and included observed richness for observed species, ACE, and Chao1 indices using *get_alphaindex* in MicrobiotaProcess (R package). The significance of differences in the measured *alpha*-diversity indices across samples were tested and visualized using nonparametric Mann-Whitney tests with *ggbox* in MicrobiotaProcess. To estimates the difference in community structure between samples, the *beta*-diversity of the samples was measured using the Bray-Curtis distance based on an evenly rarefied OTU abundance table. The method of *adonis* in vegan (R package) was used to determine the differences of the measured *beta*-diversity metrics across groups. To detect taxa with differential abundances in the groups, *diff_analysis* in MicrobiotaProcess was used, which is similar with the linear discriminant analysis (LDA) effect size (LEFSe). According to the results the Kruskal-Wallis rank sum test, Mann-Whitney test and linear discriminant analysis, with the cutoff of LDA score more than 3, the species were thought to be different between groups. The results of different analyses were visualized using ggplot2 (R packages).

**Table S1** **Primer sequences.**

| Gene | Left primer (5′-3′) | Right primer (5′-3′) |
| --- | --- | --- |
| 18s | CGATCCGAGGGCCTCACTA | AGTCCCTGCCCTTTGTACACA |
| TNF-α | CCACCACGCTCTTCTGTCTAC | AGGGTCTGGGCCATAGAACT |
| 16s | ACTCCTACGGGAGGCAGCAGT | ATTACCGCGGCTGCTGGC |
| IL-6 | TGATGCACTTGCAGAAAACA | ACCAGAGGAAATTTTCAATAGGC |
| Ccl2 | CCTGCTGTTCACAGTTGCC | ATTGGGATCATCTTGCTGGT |
| Cxcl10  Cps1  Ass1  Asl  Otc  Tjp1  Occuldin  *B.vulgatus* | CTCATCCTGCTGGGTCTGAG  CGGGAAGTAGAGATGGACGC  CGTGAAGGGGCCAAGTATGT  TGCAAGGGTTGGACAAGGTT  AGGTTACGATGAAGACTGCCA  ACCCGAAACTGATGCTGTGGATAG  CCCAGGCTTCTGGATCTATGT  GCAGATGAATTACGGTGAAAGC | CCTATGGCCCTCATTCTCAC  CCTTGGCTGATGGTCTGTGT  GGTGCCAGTGAATAGCAGGT  GCGACTTCGTCCTGTGTGTA  CTTCTGGAGCACAGGTGAGTA  AAATGGCCGGGCAGAACTTGTGTA  TCCATCTTTCTTCGGGTTTTCA  OGTCAGAGTCCTCAGCGGAAC |


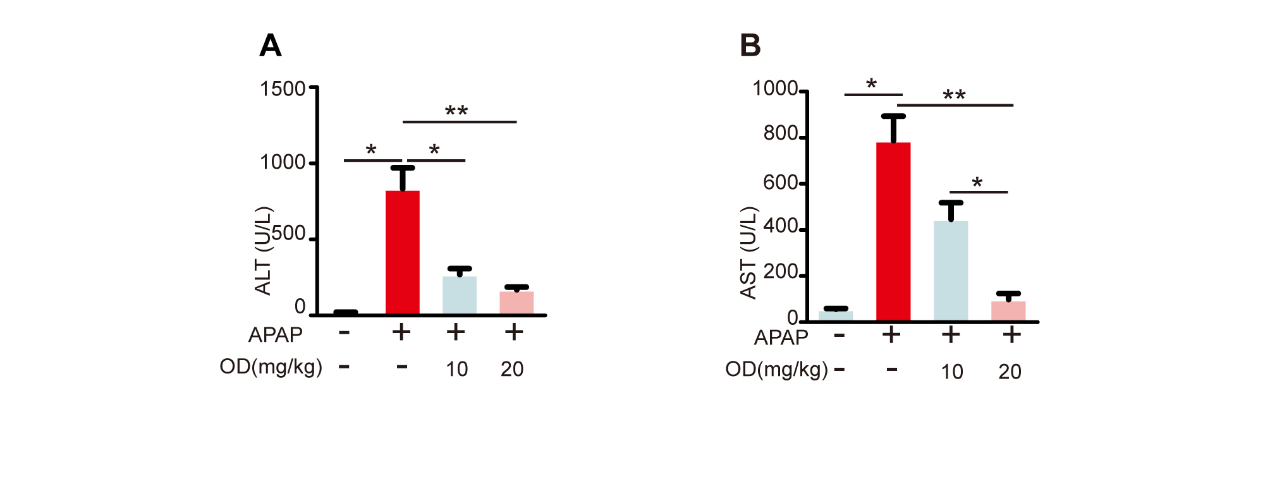


**Figure S1. OD attenuated APAP hepatotoxicity in a dose-dependent manner. (A, B)** Serum ALT and AST levels (n=5-6). The data were presented as the mean ± SEM. Statistical significance was described as * *p* < 0.05 or ** *p* < 0.01.


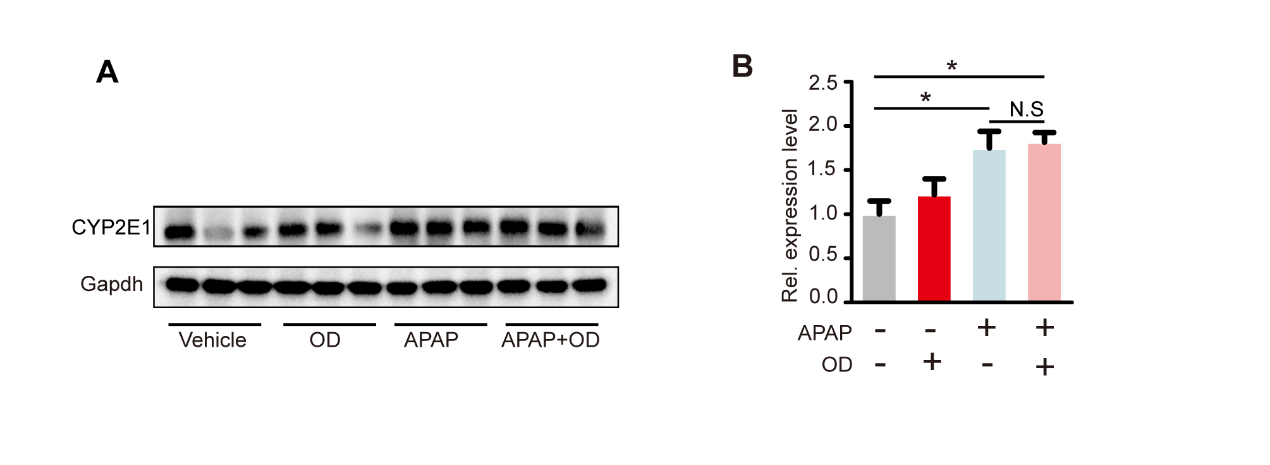


**Figure S2.** **OD does not significantly affect CYP2E1 protein expression. (A, B)** Western blot analysis of CYP2E1 (n = 3). Statistical significance was described as * *p* < 0.05. N.S: no significant.


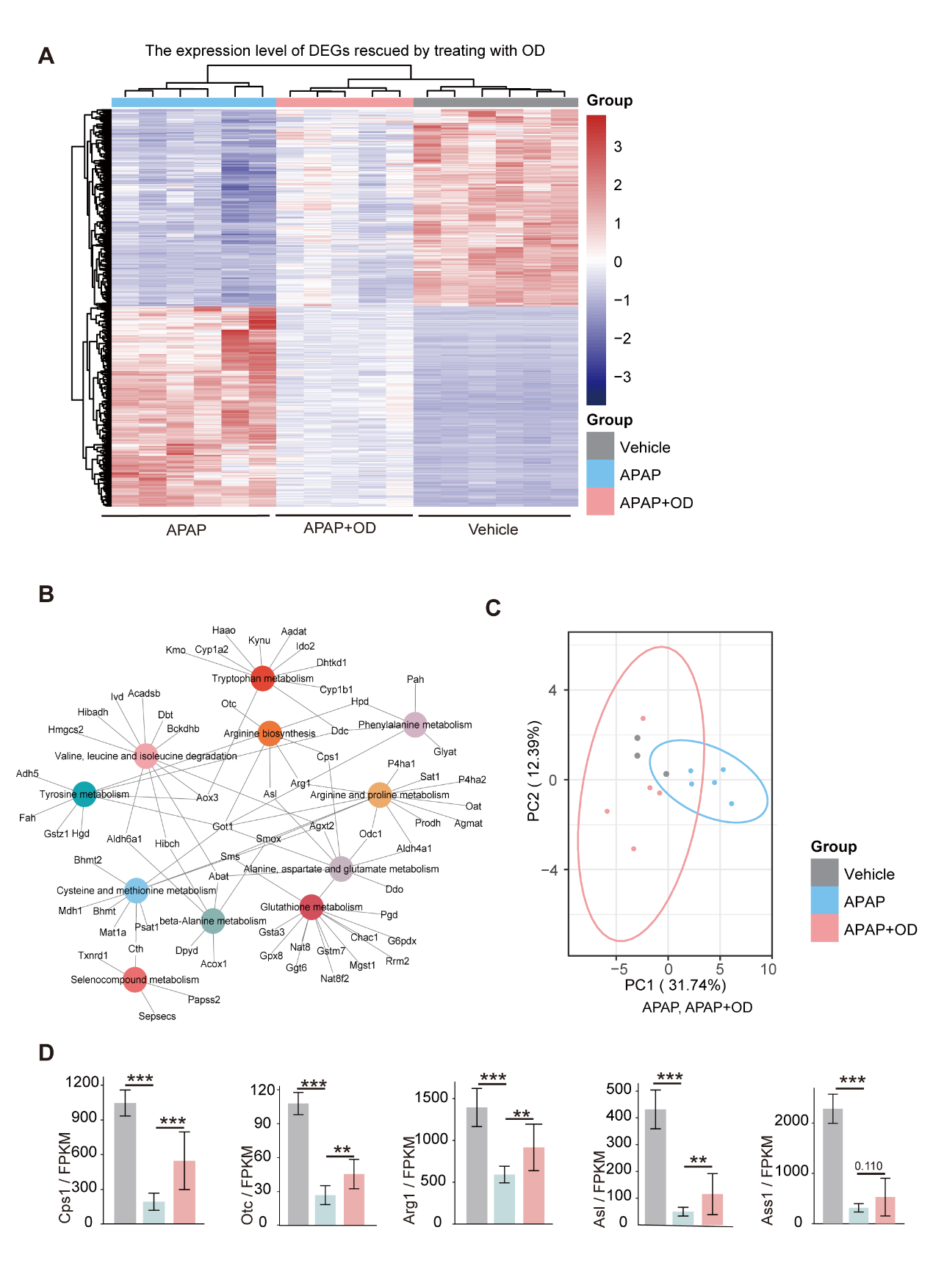


**Figure S3. OD altered amino acid metabolism dysbiosis and urea cycle dysregulation against APAP hepatotoxicity. (A)** The expression level of rescued genes in each group (n=5-6). **(B)** Network analysis of amino acid metabolism-related genes. **(C)** Principal components analysis (PCA) plot for discriminating the hepatic amino acid metabolomics (n=3-5). **(D)** Urea cycle related genes expression in RNA-seq data (n=5-6). FPKM: fragments per kilobase million, it is a normalized estimation of gene expression based on RNA-seq data. The data were presented as the mean ± SEM. Statistical significance was described as ** *p* < 0.01 or *** *p* < 0.001.


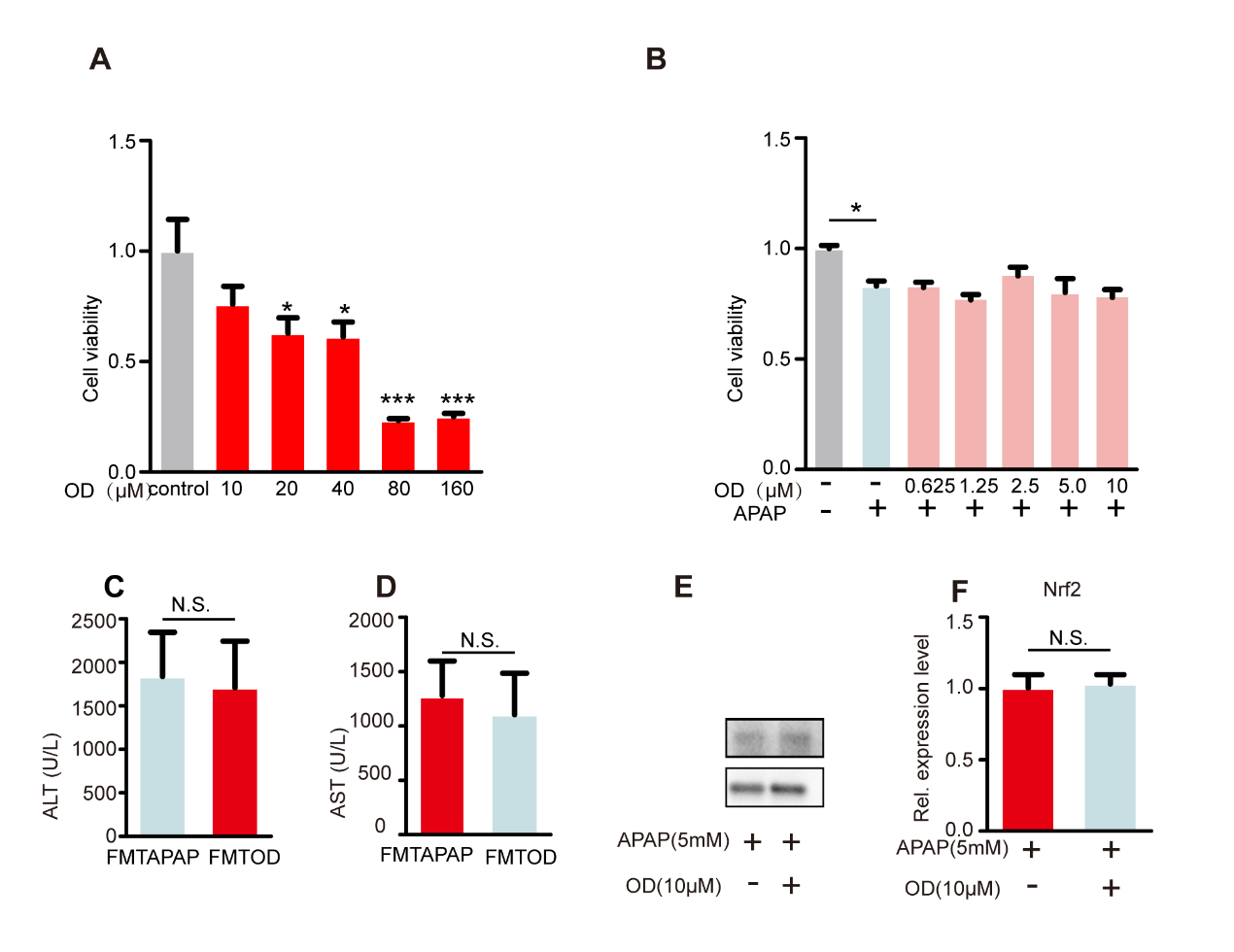


**Figure S4.** **OD could not directly suppress hepatocyte apoptosis in vitro. (A)** Human LO2 cells were incubated with different concentrations of OD for 24h. Thereafter, cell viability was tested by CCK8 assay (n=6). **(B)** Cells were co-treated with APAP (5mM) and OD for 24h. And tested cell viability (n=6). **(C, D)** FMT experiment in Nrf2-/- mice. Serum ALT and AST levels (n=5-6). **(E, F)** Western blot analysis of Nrf2 in vitro (n = 3). Statistical significance was described as * *p* < 0.05 or *** *p* < 0.001. N.S: no significant.


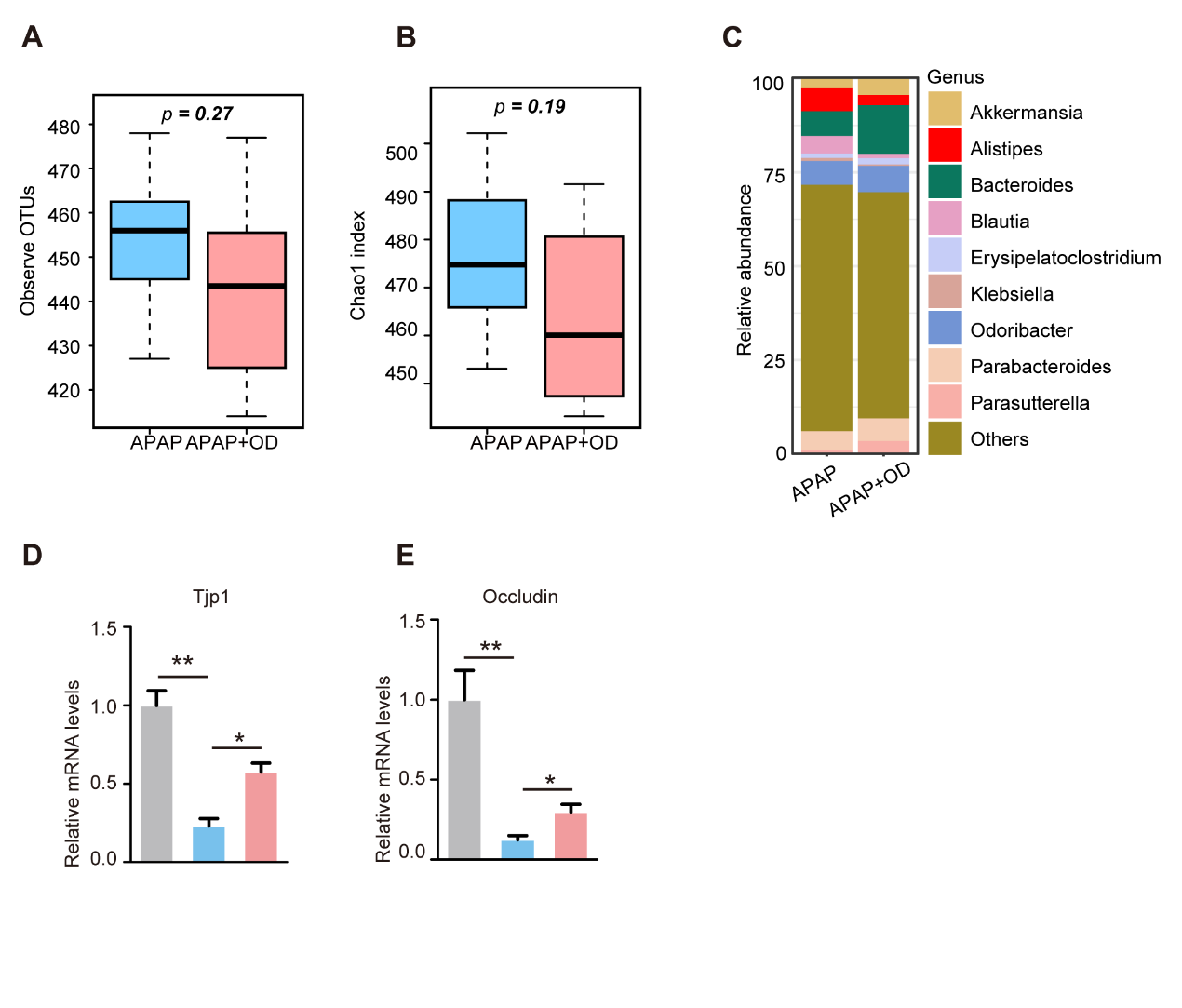


**Figure S5. OD altered gut dysbiosis against APAP hepatotoxicity. (A, B)** Comparison of *alpha*-diversity indices (observed operational taxonomic units and Chao1 Index) between each group (n=8). **(C)** The relative abundance of bacteria at the genus level (n=8). Colon mRNA levels of Tjp1**(D)** and Occludin **(E)** (n=6). Statistical significance was described as * *p* < 0.05 or ***p* < 0.01.


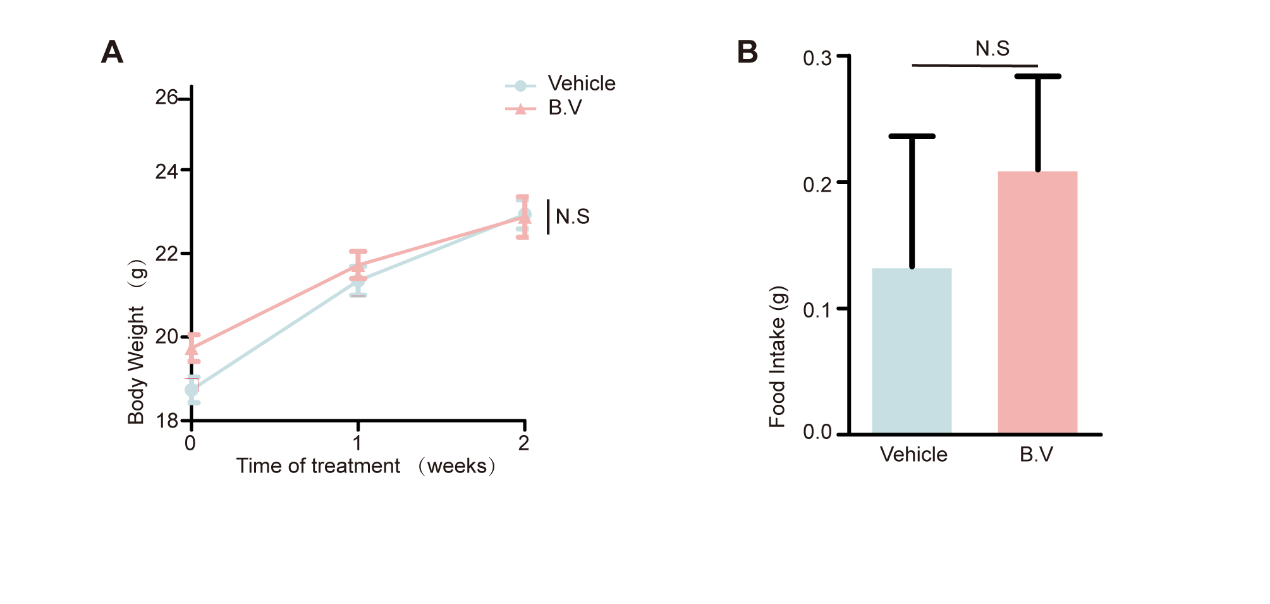


**Figure S6. Effect of *B. vulgatus* on Body weight gain (A) and food intake (B).** (n=6). The data were presented as the mean ± SEM. N.S: no significant.
